# Atypical and rare cause of myocardial infarction: coronary subclavian steal syndrome (CSSS) treated by a carotid-subclavian bypass in a 71-year-old female patient

**DOI:** 10.1186/s13019-021-01625-5

**Published:** 2021-08-21

**Authors:** Mikolaj Walensi, Johannes Bernheim, Nikodemus Ulatowski, Michal Piotrowski, Konstantinos Karaindros, Benjamin Juntermanns, Christian Mikat, Roland Heesen, Johannes N. Hoffmann

**Affiliations:** 1 Department of Vascular Surgery and Phlebology, CONTILIA Heart and Vascular Center, Elisabeth Hospital Essen, Klara-Kopp-Weg 1, 45138 Essen, Germany; 2 Department of Cardiothoracic Surgery, Medical University of Gdansk, Gdansk, Poland; 3 Department of Emergency Medicine, William Osler Health System, Brampton, ON Canada; 4 Department of Radiology, Elisabeth Hospital Essen, Essen, Germany; 5 Department of Angiology, CONTILIA Heart and Vascular Center, Elisabeth Hospital Essen, Essen, Germany

**Keywords:** Coronary subclavian steal syndrome, Carotid-subclavian bypass, Coronary arterial bypass graft operations

## Abstract

**Background:**

The coronary subclavian steal syndrome (CSSS) is a rare complication after coronary arterial bypass graft operations (CABG) using the left or right internal mammary artery ((L/R)IMA). It results from a retrograde blood flow from the IMA into the subclavian artery (SA) due to a stenosis or occlusion of the SA proximal to the IMA origin. This “steal phenomenon” leads to a decreased blood flow in the IMA and may result in myocardial ischemia (MIS) and even myocardial infarction (MIN). Treatment options include interventional and surgical therapy.

**Case presentation:**

We report the case of a 71-year old woman, who suffered from an acute non-ST elevation myocardial infarction (NSTEMI) 11 years after LIMA-CABG surgery and who was treated successfully with a carotid-subclavian bypass (CSB) after failed interventional therapy.

**Conclusion:**

CSB may be regarded as a viable treatment option for patients suffering CSSS in the case of MIS and even an acute MIN/NSTEMI, especially in the case of missing or failed interventional therapy attempts. Specialists in cardiothoracic and vascular surgery should be aware of possible CSSS conditions and know about appropriate diagnostic and therapeutic options.

## Background

The coronary subclavian steal syndrome (CSSS) is a rare complication after coronary arterial bypass graft surgeries (CABG) using the left or right internal mammary artery ((L/R)IMA, also internal thoracic artery) [1–3]. It results from a retrograde blood flow from the IMA into the subclavian artery (SA) due to a stenosis or an occlusion of the SA, proximal to the IMA origin [1, 2, 4]. This “steal phenomenon” leads to decreased blood flow in the IMA and may lead to myocardial ischaemia (MIS) and even myocardial infarction (MIN, (non-)ST elevation myocardial infarction ((N)STEMI)) [1, 2, 5]. Patients may be completely asymptomatic [1, 6], present with unspecific symptoms like dizziness, syncope or dyspnea [7], or specific symptoms of the upper limb (arm pain, claudication, numbness, coldness, paraesthesia, weakness) [3] or myocardial (recurrent angina, chest pain, cardiac insufficiency) [1] ischaemia with signs of an acute MIN (ST-elevation in the electrocardiogram (ECG), cardiac enzyme elevation) [8, 9].

The diagnosis may be provided by Doppler and/or duplex sonography, CT- and/or MR-angiography (CT-A, MR-A), or digital subtraction angiography (DSA) [1, 4, 10, 11]. Treatment options include interventional procedures as well as numerous surgical options [1, 12]. Here, we report the rare case of a patient with CSSS, suffering from an NSTEMI, which failed interventional treatment and therefore required surgery.

## Case presentation

We report the case of a 71-year old female patient (165 cm, 83 kg) who was transferred to our department for vascular surgery with a CSSS-related acute NSTEMI in 05/2017. The patient underwent twofold CABG (LIMA to Ramus circumflexus (RCX), RIMA to RIMA/LAD) in 08/2006. Beside coronary heart disease, she suffered from bradycardia and atrial fibrillation, arterial hypertension, type II diabetes mellitus, hypercholesterolaemia, obesity, Fountain’s stage IIa peripheral arterial occlusive disease (PAOD), pulmonary hypertension, stage III chronic kidney disease, and hepatitis B. According to her medical history, she has undergone multiple interventional therapies for the PAOD and an internal carotid artery (ICA) thrombendarterectomy due to an ipsilateral stenosis in 10/2005 and an occlusion of the right ICA. A coronary angiography (CA) in early 2017 revealed no pathologies with both CABGs open and functioning.

**Fig. 1:**
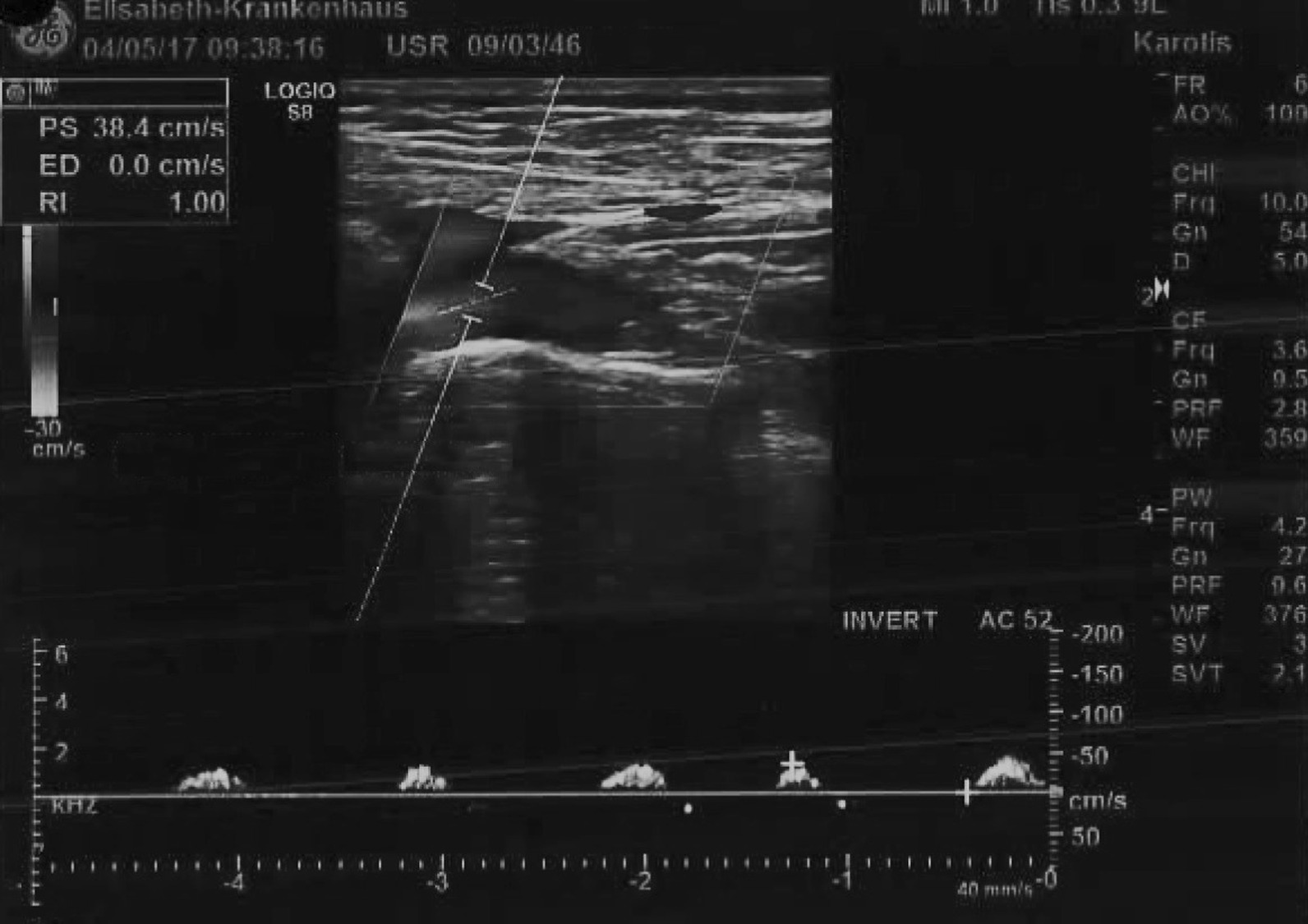
Sonography of the left proximal subclavian artery, showing a low flow profile (0.38 m/s) with the subsequent occlusion

**Fig. 2:**
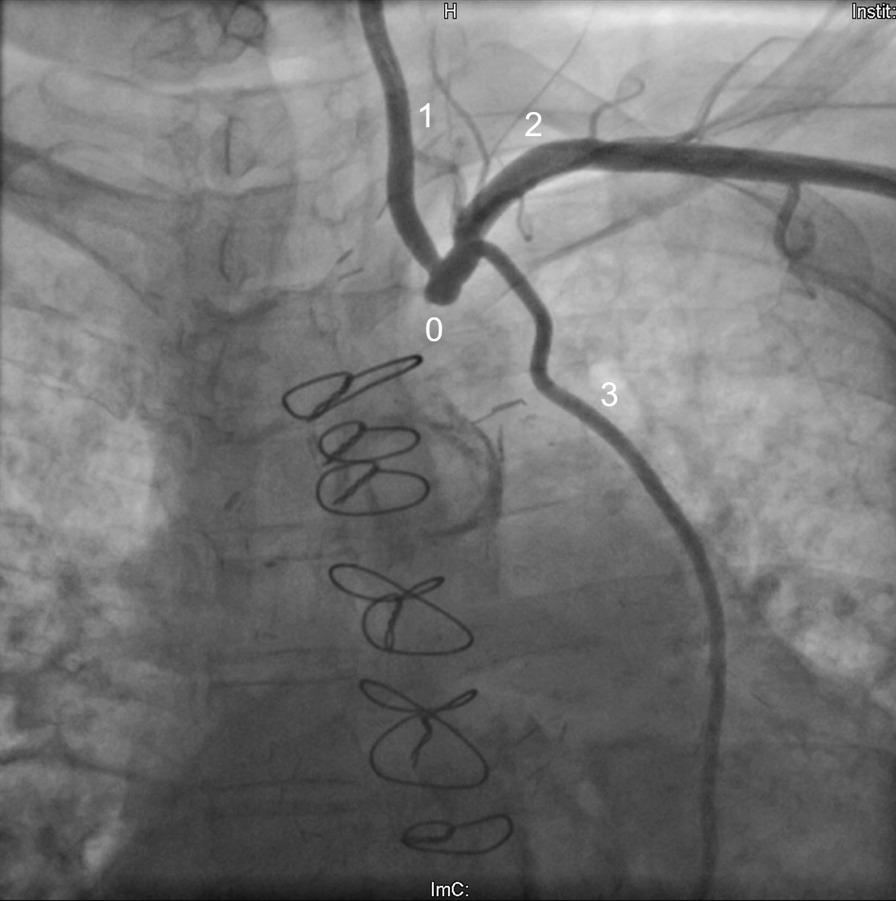
Angiography of the left supraaortic arteries, showing the proximal left subclavian artery occlusion (0), the left common carotid artery (1), the left subclavian artery (2, SA), and the patent coronary artery bypass graft, performed of the left internal mammary artery (3). The occlusion of the SA could not be passed with the guide wire during the procedure. Therefore, an interventional therapy of the occlusion was impossible. A coronary angiography was performed simultaneously during the same angiographic session (Fig. 3)

**Fig. 3:**
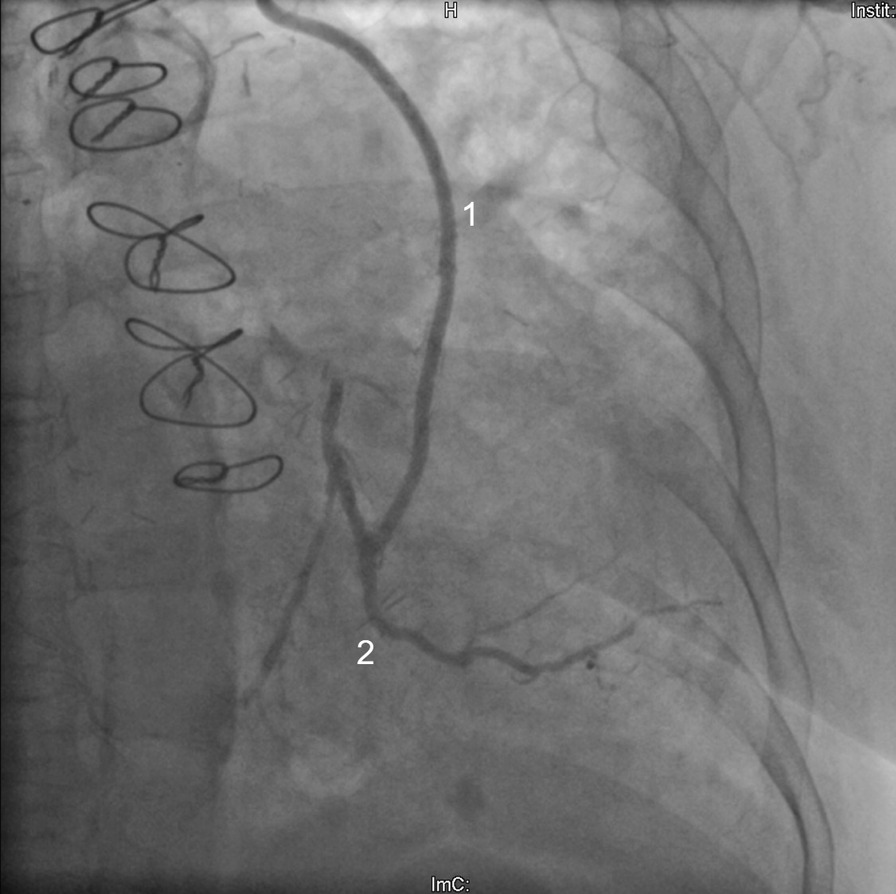
Coronary angiography, showing the patent coronary artery bypass graft, performed of the left internal mammary artery (1) and the circumflex artery. An angiography of the left supraaortic branches was performed simultaneously during the same angiographic procedure (Fig. 1)

**Fig. 4:**
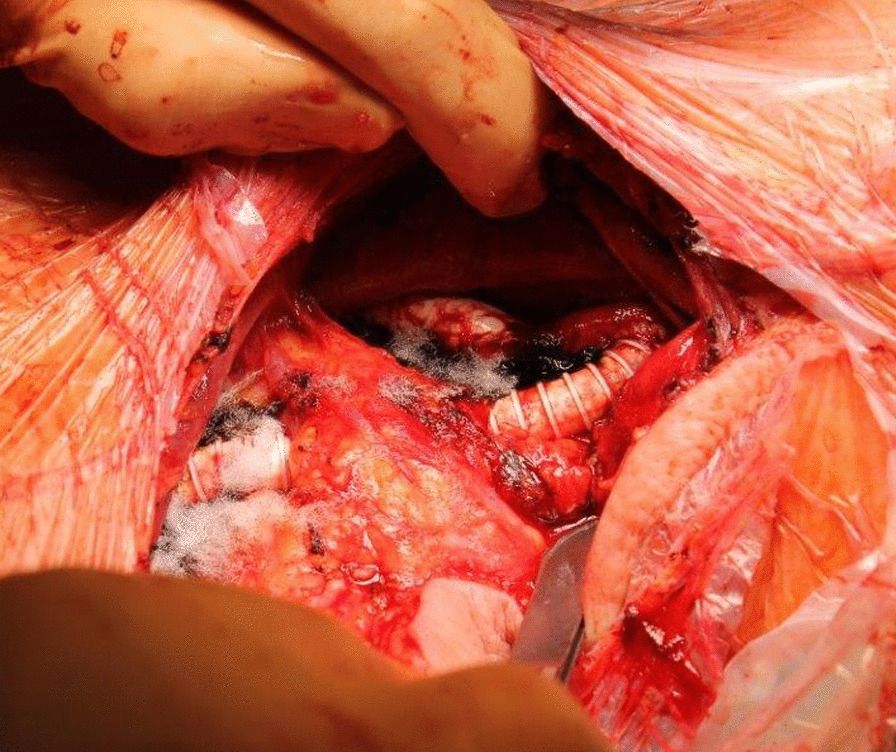
Intraoperative view, showing the subcutaneous course of a carotid-subclavian bypass (polytetrafluoroethylene (PTFE), 8 mm, ring reinforced)

**Fig. 5:**
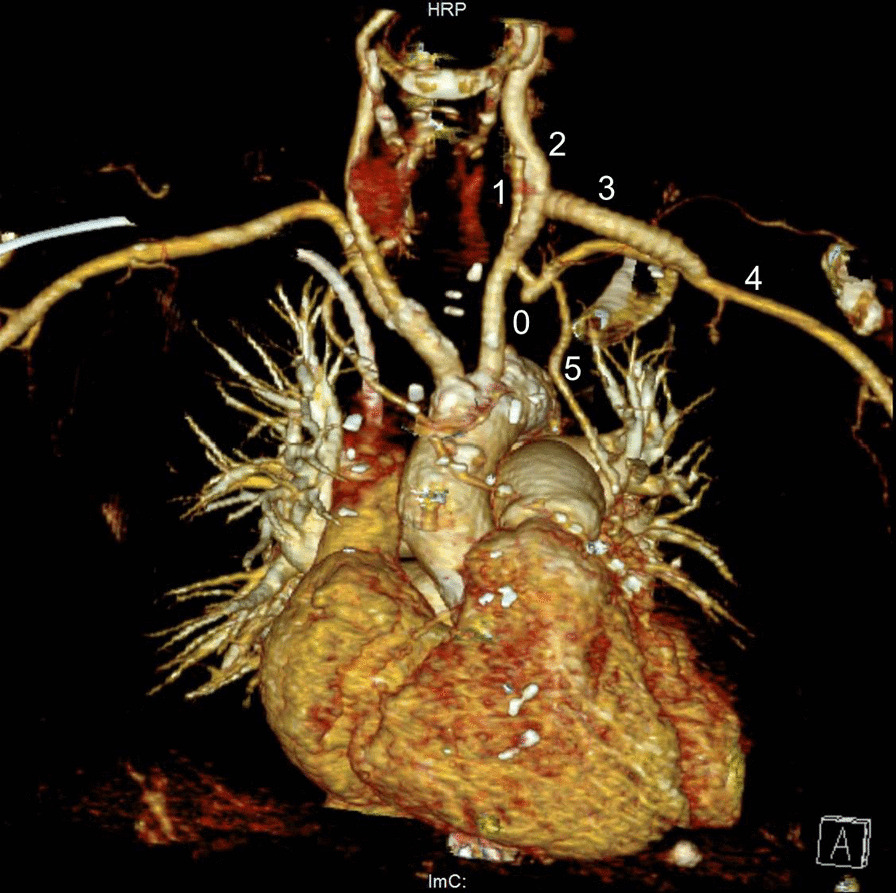
3D image reconstruction of the postoperative CT-angiography control, showing the vascular reconstruction with the proximal left subclavian artery occlusion (0), the left vertebral artery (1), the left common carotid artery (2), the carotid-subclavian bypass (3), the left subclavian artery (4), and the patent coronary artery bypass graft, performed using the left internal mammary artery (5)

The initial cardiological presentation in late 04/2017 took place in the emergency department due to recurrent syncopes, bradycardia and chest pain after exhaustive left arm strain. Beside a sinus bradycardia, a NSTEMI with elevated troponin levels of 0.45 ng/ml (0.27 ng/ml the following day, normal values < 0.014) was diagnosed. Occlusion pressures (mmHg) were 60 in the left and 130 in the right arm with a systemic blood pressure of 120/60. Duplex sonography showed a proximal occlusion of the left SA and retorgrade flow in the ipsilateral vertebral artery (Fig. 1). Two arterial punctures (transfemoral and transbrachial, both from the left side) and a CA were performed. Both CABGs proved to be patent and without stenoses (Figs. 2 and 3) and therefore a CSSS was assumed to be the cause of the NSTEMI. Two attempts for an interventional recanalization of the left SA failed as the SA occlusion could not be passed with the guide wire due to severe calcifications. Subsequently, the patient was transferred to our department for vascular surgery for further diagnostics and therapy in early 05/2015. Here, a CT-A was performed to evaluate the vascular configuration and proved the SA stenosis as well as the CSSS as the underlying pathologies. Within our interdisciplinary vascular conference, we agreed on the indication for bypass surgery. A left-sided carotid-subclavian bypass (CSB) (polytetrafluoroethylene (PTFE) prosthesis, 8 mm, ring reinforced) was performed (Fig. 4). Postoperatively, the occlusion pressures were 120 in the left and 120 in the right arm with a systemic blood pressure of 120/80. Duplex sonography showed a regular antegrade blood flow in the CSB and a CT-A proved a regular configuration and patency of the CSB and both CABGs (Fig. 5). A cardiac adenosine-stress-MR showed a perfusion deficit/hypoperfusion in the supply area of the left and right coronary artery and ischaemic scar tissue of the basal and anterior medial and posterior cardiac wall. No postoperative complications occurred; especially no chest pain, dyspnea, syncope or symptoms in the left arm were observed. Low molecular weight heparin (enoxaparin sodium) was administered in a therapeutic dosage (80 mg s.c, twice daily) and an anticoagulative therapy with phenprocoumon was recommended with a target-INR of 2–3. Additionally, Clopidogrel 75 mg orally was prescribed once daily. The patient was discharged on the 10th postoperative day in a good general condition. At the follow-up-visits at 6 weeks, 6 months and 12 months postoperatively, the patient was in a good general condition without cardiac or neurological symptoms. The patency of her bypass graft was demonstrated on duplex sonography on each with these visits.

## Discussion

The CSSS is a rare complication after CABG using the IMA, first described by Harjola and Valle in 1974 [13]. The incidence varies between 0.07 and 3.4%, early and late onsets (2–31 years after CABG) have been described [10]. The most common cause is a stenosis or an occlusion of the left SA proximal to the origin of the LIMA graft, leading to a flow reversal and MIS after strain of the left arm [10].

Due to an incidence of SA stenoses of up to 2.7% in patients requiring CABG surgery and the increased use of the IMA as a CABG-conduit, a standardized preoperative screening of these patients for SA stenoses before undergoing CABG surgery using the IMA has been repeatedly recommended [1, 11, 12]. In the case of a SA stenosis, a simultaneous therapy performing the CABG and an interventional or surgical therapy for the SA lesion should be considered [10]. The clinical presentations vary from an asymptomatic steal phenomenon to silent ischemia [1, 10, 12, 14], unspecific cardiac symptoms [4], rarely different forms of MIN [8] or even heart failure [10]. Our patient suffered from a manifest NSTEMI with elevated troponin levels and clinical symptoms. The SA stenosis may also lead to symptoms in the arm after exhaustive strain, including all symptoms of extremity ischemia [1, 12], which were also present in our patient. Diagnostic procedures include bilateral blood pressure measurements (in case of physiological measurements also with careful provocative studies), as well as Doppler and duplex sonography of the SA [1, 8, 15]. To evaluate the vascular configuration as well as the presence of a stenosis or occlusion and to validate the patency of the arteries, a CT- or MR-A of the aortic arch and its branches might be performed [1, 8]. Angiography remains the gold standard as it may be performed together with an additional CA. It can prove the patency of the CABG and allows a simultaneous therapeutic intervention of the SA pathology [8]. Regarding the therapeutic options, interventional procedures like plain old balloon angioplasty (POBA), stent implantation [15, 16], cutting balloon or laser [1] as well as various open surgical approaches like the performance of a CSB [1, 12], an aorto-subclavian bypass [1], a carotid-axillary bypass [10], an axillo-axillary bypass [1], a subclavian-to-carotid transposition [17], a subclavian-subclavian bypass [17, 18] or a re-insertion of the IMA-graft into the aorta [1] have been described. In case interventional therapy is not an option or a failed interventional therapy attempt, mainly due to severe calcifications of the SA, bypass surgery is required [1, 19]. That is even the case in sick patients with severe CSSS symptoms, where an interventional therapy was attempted initially [4]. Even if most CSSS might be treated interventionally, a few reports, including our case, suggest that bypass surgery is possible even in advanced stages of CSSS as demonstrated in our patient, who suffered a NSTEMI [4, 10]. Regarding our approach, it is *crucial* to rule out an ICA stenosis before performing a CSB to prevent cerebral ischemia [12]. Regarding the surgical treatment of the CSSS in general, excellent long term results might be achieved while 5-year re-stenosis rates of POBA and stent therapies reach 41% and 16%, respectively [10].

## Conclusion

In our case, an advanced stage of the CSSS has been successfully treated with a CSB after failed interventional therapy, proving CSB to be a safe, feasible and effective therapeutic option. A CSB may therefore be regarded as an excellent primary treatment option for patients suffering from CSSS, even in the case of an acute MIN and especially in the case of lacking or failed interventional therapy attempts as a secondary treatment option. Moreover, we believe that knowledge about CSSS is crucial for cardiothoracic and vascular surgeons as in patients with a SA stenosis, who are undergoing CABG surgery as this may prevent iatrogenic CSSS and might as well necessitate to adjust the initially chosen therapeutic option for such patients.

## Data Availability

All data generated or analyzed during this study are included in this published article.

## Abbreviations

CSSS: Coronary subclavian steal syndrome
CABG: Coronary arterial bypass graft
(L/R)IMA: Left or right internal mammary artery
SA: Subclavian artery
MIS: Myocardial ischemia
MIN: Myocardial infarction
CSB: Carotid-subclavian bypass
PAOD: Peripheral arterial occlusive disease
ICA: Internal carotid artery
POBA: Plain old balloon angioplasty
